# In vitro investigation of *Mangifera indica* L. peel extracts: antibacterial, antioxidant, and docking studies

**DOI:** 10.1186/s13568-025-01882-w

**Published:** 2025-05-08

**Authors:** Salwa M. El-Sayed, Basma T. Abd-Elhalim, Khalid A. El-Dougdoug, Rawia F. Gamal, Ghada G. El-Bana, Nesma Nabil Ibrahim Mohamed

**Affiliations:** 1 Department of Biochemistry, Faculty of Agriculture, Ain Shams University, PO Box 68, Hadayek Shoubra,Cairo, 11241 Egypt; 2 Department of Agricultural Microbiology, Faculty of Agriculture, Ain Shams University, Hadayek Shoubra, Cairo, 11241 Egypt; 3 Department of Chemistry, Faculty of Science, Mansoura University, El-Gomhoria Street, Mansoura, 35516 Egypt; 4 Mansoura University Student’s Hospital, Mansoura University, El-Gomhoria Street, Mansoura, 35516 Egypt

**Keywords:** Antibacterial, Antibiofilm, Antioxidant activity, Colon cancer, Mango peels, Phytochemical screening

## Abstract

Marketers of antioxidants and antimicrobials view fruit peels as a special, readily available, reasonably priced, natural, eco-friendly, and lucrative source. Mangos (*Mangifera indica* L.) and their byproduct peels and kernels are nutrient-dense, distinctive, affordable, efficient, natural, and environmentally friendly sources of antibacterial agents, antioxidants, and other active chemicals. The current study aimed to prepare extracts of different degrees of polarity from mango peels, detect their active phytochemical compounds, and study their effects as antioxidants, antibacterials against food-borne pathogens, anti-biofilms, and anti-colon cancer in vitro. Phytochemical classes of compounds were screened using different standard methods. The most promising profile was for mango peel ethyl acetate extract (MPEE) due to the presence of a variety of phytochemicals including tannin, coumarin, alkaloids, flavonoids, phenols, steroids, and terpenoids at high concentrations compared to other mango peel extracts. Therefore, it was selected as the most valuable extract to examine its anti-colon cancer impact, anti-foodborne pathogenic bacteria, and antibiofilm. The anti-foodborne pathogenic activity of MPEE was evaluated against *Enterococcus faecalis* ATCC 7080, *Staphylococcus aureus* ATCC 5638, *Salmonella typhi* DSM17058, *Bacillus cereus* ATCC 11778, *Shigella sonnei* DSM 5570, and *Escherichia coli* ATCC 8739 that showed highly impact for all. At MIC and MBC values of 500 μg/ml, the MPEE had a 100% bactericidal spectrum; at lower concentrations of 125–250 µg/ml, no antibacterial action was seen. The MPEE had a biofilm inhibition percentage ranging between 98.75–53.33%; *B. cereus* had the highest percentage and *S. sonnei* had the lowest. Furthermore, The MPEE demonstrated an anticancer activity against human colon epithelium ATB-37 (Caco2) with an IC_50_ of 430.36 µg/ml. Molecular docking modeling assessment illustrated top-ranked confirmations between major phytochemicals and target protein COX2: P00406 and NFKB: Q63369.

**Supplementary Information:**

The online version contains supplementary material available at 10.1186/s13568-025-01882-w.

## Key points

- Mango peels ethyl acetate extract (MPEE) has varied active compounds with different bioactivities.
- MPEE has high antibacterial action for various food borne pathogen bacteria.
- MPEE has anticancer effect on Human colon epithelial carcinoma ATB-37 (Caco2) with IC_50_ of 430.36 μg/ml.

**Supplementary Information:**

The online version contains supplementary material available at 10.1186/s13568-025-01882-w.

## Introduction

The advent of resistant antibiotics in some food-borne microbes and consumer reluctance to consume chemically treated commodities have fueled the invention of safe and natural antifood-borne pathogens for food products and the management of various diseases (Okareh et al. 2019; Noshad et al. 2022; Jalil et al. 2023). Approximately half of all human deaths are caused by infectious diseases, of which microbes are the most common cause. Infections are caused by a variety of bacterial etiologic agents, such as pathogenic *E. coli*, *Bacillus cereus*, *Staphylococcus aureus*, and *Salmonella typhi*. are most prevalent (Diso et al. 2017; Noshad et al. 2023; Mosallaie et al. 2024). Diarrhea-related morbidity and death are also a substantial problem in many poor nations.

Fruit peels are regarded as a unique, easily accessible, affordable, environmentally friendly, natural, and profitable source for marketers of antioxidants and antimicrobials (Sarker et al. 2017). Mango peels are considered to be a special, easily accessible, cost-effective, natural, and environmentally beneficial source of antibacterial and antioxidant compounds (Ogidi et al. 2021; Alizadeh et al. 2024). The mango (*M. indica* L.), a fruit belonging to the Anacardiaceous family, is one of the most extensively farmed fruits in the world. Companies produce up to several million tons of mango trash every year. (Bshabshe et al. 2020; Alizadeh et al. 2025). About 15–20 percent of the fruit is typically thrown away as trash, with the peel which is thought to be a by-product of industrial production or fruit consumerism making up this portion (FAO 2017).

Mango peels have garnered significant interest from scientists lately because of their abundance of beneficial compounds, including enzymes, carotenoids, phytochemicals, polyphenols, and vitamins E and C, which contain important functional and antioxidant qualities (Maharaj et al. 2022). In addition, mango peels are abundant in dietary fiber, lipids, protein, enzymes, cellulose, and hemicellulose, as well as pectin (Ningsih et al. 2019). The health of humans benefits from these essential substances as well. Mango peels are utilized in the creation of priceless components (Spizzirri et al. 2021).

Nowadays, mango peel flour is a helpful component of many various foods, such as sponge cakes, biscuits, bread, noodles, as well as other baked items. There is a severe disposal issue as a result of ongoing efforts to find better ways to use leftover fruits and vegetables (Espinosa-Espinosa et al. 2022). The repurpose of biological wastes is highly relevant as industry is increasingly compelled by law and environmental concerns to find a new use for its leftover material, such as seeds and peels (Poomanee et al. 2020). In addition to assessing the in-vitro antioxidant, antifood borne pathogen bacteria, antibiofilm, and anticancer characteristics of the most promising mango peel extract, the current study utilized GC–MS and HPLC chromatography to assess the key phytochemicals.

## Materials and methods

### Chemicals, media and reagents

The DPPH regent, Folin-Ciocalteu’s reagent, and gallic acid and quercetin standards were provided by Sigma-Aldrich. Mueller Hinton agar with code PO1191 and Nutrient Agar with code CM0003B media were purchased from OXOID, UK. All of the chemicals used in this investigation were analytically accurate.

### Collection of *M. indica* L. peels

As mentioned by Thambi et al. (2016) Fresh mangoes were obtained from a local market that were bright golden in colour and free of bruises. The fruits were laved by running water and patted dry with a cleansed cloth before being peeled. We used a sharp knife that had been sanitized to remove the peels. One kilogram of mangos yielded, on average, 200 g of peel.

### Food borne pathogenic microorganisms

Six strains of food-borne pathogens, *Enterococcus faecalis* ATCC 7080, *Staphylococcus aureus* ATCC 5638, *Salmonella typhi* DSM17058, *Bacillus cereus* ATCC 11778, *Shigella sonnei* DSM 5570, and *Escherichia coli* ATCC 8739. The strains were generated from the Microbiology Dep., Fac. of Agric., Ain Shams Univ. For antimicrobial activity assays, the foodborne pathogen strains were cultured overnight at 37 °C in nutrient broth medium after being maintained and preserved in nutrient agar medium at 4 °C.

### Food borne pathogens standard inoculum preparation

The food borne pathogens standard inocula were prepared according to (Ballesteros-Vivas et al. 2019; Galal et al. 2021). A loop of a Freshly created culture of food-borne pathogens inoculum was implanted into 50 ml of nutrient broth medium and underwent incubation for 24 h at 37 °C at 150 rpm under shaking (Shin Saeng- South Korea).

### Mango peel extracts (MPE) preparation

Preparation of MPE were established as described by Mantilla-Mantilla et al. (2021). Lyophilized mango peels (100 g) were taken out with 500 ml of four solvents (EtOH, BuOH, CH_3_Cl and CH_3_COOC_2_H_5_) for 24 h. at 4 °C. After that, the extracts underwent centrifugation (SIGMA 3–16 K, Germany) at 6000 rpm for 15 min. The residue was extracted 3 times, then the supernatants were vacuum evaporated at 40 °C in a rotating evaporator (RE-102, USA). The residual extracts underwent lyophilization (Hull 24 sq.ft., Lyolab, New Jersey, US) for phytochemical investigations.

### Phytochemicals content investigation of MPE

Different MPE of ethanolic, butanol, chloroform and ethyl acetate extracts were subjected to qualitative analysis of phytochemicals (Ballesteros-Vivas et al. 2019; Elakraa et al. 2022). Standard screening assays were performed on MPE to identify Phytoconstituents. Standard techniques were applied for assessing several metabolites, including tannins, flavonoids, saponins, steroids, cardiac glycosides, phlobatannins, alkaloids, and phenolics (Table S1).

### Quantitative analysis of some phytochemicals of MPEE

#### Total phenolic compounds (TPC) investigation

Folin Ciocalteu’s reagent and 20% Na_2_CO_3_ were used to determine TPC, and a microplate reader (BioTek Instruments Inc., Bad Friedrich shall, Germany) was used to measure absorbance at 725 nm (El-Batal et al. 2019). The data were presented as μg gallic acid equivalents (GAE)/g dry weight (DW) on a dry weight basis after three replicate analyses.

#### Total flavonoids content (TFC)

TEC was estimated with the Na_2_NO_2_ (5%)/AlCl_3_ (10%)/NaOH (1 M) performance establishing the absorbance at 510 nm (Ballesteros-Vivas et al. 2019). The results were given as μg of Quercetin equivalents (CE)/g dry weight (DW). In addition, a triplicate analysis was conducted (n = 3).

#### Ascorbic acid content (AAC)

The 2,6-dichlorophenol indophenols titration method, as outlined by AOAC, was used to measure the AAC level (2000) (van de Loosdrecht et al. 1994; Ballesteros-Vivas et al. 2019).

#### MPEE phenolic compounds separation by high-performance liquid chromatography (HPLC)

A volume of 5 μl of each sample solution was injected in Agilent 12,060 series HPLC with an Eclipse C18 column (4.6 × 250 mm i.d., 5 μm) at 40 °C for separation of phenolic compounds. Also, the mobile phase was composed of H_2_O (A) and 0.05% CF_3_COOH (Sigma-Aldrich, Merck, Germany) in CH_3_CN (B) (HPLC-grade 99.9%) at a flow rate of 0.9 ml/min. Furthermore, the mobile phase was produced using a linear gradient as seen below. The following intervals were used: 0 min (82% A), 0–5 min (80% A), 5–8 min (60% A), 8–12 min (60% A), 12–15 min (82% A), 15–16 min (82% A), and 16–20 min. Moreover, at 280 nm, the multi-wavelength detector was inspected (Morris et al. 2009).

#### Gas chromatography mass spectrometry (GC–MS) analysis of MPEE

The sample was extracted twice using ethyl acetate as the solvent. A 1:1 (v/v) ratio of solvent to the filtered sample was mixed and shaken vigorously for 20 min. Using a separating funnel, the bioactive components in the ethyl acetate layer were extracted from the aqueous phase. This layer was then concentrated by evaporation to dryness at 50 °C. The resulting residue was purified with methanol and prepared for GC–MS analysis according to the method described by Ahsan et al (2017). Moreover, a TG-5MS capillary column (30 m × 0.25 mm × 0.25 μm film thickness) and a Trace GC-TSQ mass spectrometer (Thermo Scientific, Austin, TX, USA) were utilized. The column temperature was initially set at 50 °C, then increased at a rate of 5 °C/min to 250 °C, where it was held for 2 min. Subsequently, the temperature was raised at 30 °C/min to a maximum of 300 °C and maintained for 2 min. The injector temperature was set at 270 °C, and the MS transfer line was maintained at 260 °C. Helium was used as the carrier gas at a steady flow rate of 1 mL/min. Using the auto sampler AS1300 in split mode, 1 μL of diluted sample was automatically injected, with a solvent delay of 4 min. The EI mass spectra were acquired at an ionization voltage of 70 eV, scanning across an *m/z* range of 50–650 in full scan mode. The ion source temperature was maintained at 200 °C. Component identification was performed by comparing the obtained mass spectra with those in WILEY 09 and NIST 14 mass spectral databases (Maamoun et al. 2021).

#### Antioxidant activity assessment of MPEE

The DPPH radical scavenging capacity was used to assess MPEE’s antioxidant activity. (Youl et al. 2023). In this method, 300 μL of the sample was mixed with 500 μL of 50 μM DPPH solution in 100% ethanol and incubated in the dark for 30 min. The absorbance of the mixture was measured at 517 nm. The percentage inhibition of radical scavenging activity was calculated using the following formula:

1 $$\% Inhibition = \left[ {\frac{{Abs.control {-} Abs.sample}}{Abs.control}} \right] \times 100 $$

where Abs control is the reaction’s absorbance in the absence of a sample.

Abs sample is a sample’s absorbance.

#### Anti-foodborne pathogenic bacteria investigation of MPEE

It was employed using the well-diffusion method (Villacís-Chiriboga et al. 2020), using a 9.0 mm diameter sterilized cork porer to make wells through the Muller Hinton agar (MHA) layer. Additionally, 50 μl of standard microbial inoculum cultures (10^6^ CFU/ml) were inoculated and evenly spread on the petri dish, followed by the addition of sterile MHA medium to each sterile petri dish. Moreover, 50 μl of 1000 μg/ml MPEE and standard antibiotics, including Streptomycin for (G + ve) bacteria and Ampicillin for (G-ve) bacteria, were added to each well. The wells were then incubated at 37 °C for 24 h, and the inhibition zones were measured in millimeters. Each plate was incubated under the same conditions for consistency. Finally, activity index (AI) was computed as follows (Campos et al. 2020; Villacís-Chiriboga et al. 2020):

2 $$\begin{aligned}AI = {{Inhibition~\;zone\;~diameter\;~by~\;MPEE}}/\\{{Inhibition~\;zone\;~diameter~\;by~\;the~\;standard\;~antibiotic}} \end{aligned}$$

#### Minimum inhibitory concentration (MIC) determination

The MIC refers to the lowest concentration of an antimicrobial agent needed to inhibit the visible growth of a microorganism. Additionally, diagnostic labs depend on their values to verify microbial resistance to antimicrobial drugs and assess the effectiveness of novel antimicrobial compounds. Moreover, the MICs were determined following the protocols outlined by Galal et al. (2021) and Mantilla-Mantilla et al. (2021). Following the preparation of two-fold serial dilutions of MPEE (0.5, 0.25, and 0.125), the end concentrations were 1000 μg/ml (control), 500 μg/ml, 250 μg/ml, and 125 μg/ml. This was achieved by transferring 1 ml from the stock solution into the first tube containing 1 ml of distilled H_2_O, followed by successive 1 ml transfers to subsequent tubes. As previously stated, a calibrated micropipette was used to transfer these dilutions into wells created in inoculated plates, which were subsequently inoculated into MHA agar wells and incubated for 24 h at 37 °C.

#### Minimum bactericidal concentration (MBC) determination

The Minimum bactericidal concentration (MBC) is determined by assessing the lowest antimicrobial concentration that kills 99.9% of the initial bacterial population. This is typically done by subculturing samples from the Minimum inhibitory concentration (MIC) test. Specifically, samples from plates showing no visible growth in the MIC assay are inoculated into fresh, antimicrobial-free MHA medium. The MBC is identified as the lowest concentration of the antimicrobial agent where no growth is observed. This indicates that at this concentration, the antimicrobial agent has effectively killed the bacteria rather than merely inhibited their growth (Galal et al. 2021; Mantilla-Mantilla et al. 2021).

#### Antibacterial activity mode of action

According to MIC and MBC obtained results, the MBC/MIC ratio was calculated. The MBEE had a bactericidal effect when the ratio’s value was equal to or larger than 4. Conversely, static action is defined as ratio values of 2 or less (Mantilla-Mantilla et al. 2021).

3 $$\begin{aligned}Antimicrobial\,~agent\,~mode\,of\,~action =\\{{Minimum\,bactericidal~\,concentration\,\left({MBC} \right)}}/\\{{Minimum\,~inhibition\,~concentration\,\left( {MIC} \right)}} \end{aligned}$$

#### Antibiofilm activity of MPEE

Following the test tube’s assay against the chosen food-borne pathogenic bacteria, the findings were compared with the control, untreated samples, and lastly, the semi-qualitative assay concerning the microbial biofilm hindrance. This allowed for the evaluation of the MPEE’s antibiofilm potential (Elakraa et al. 2022). Prior to the antibiofilm assay, the tested bacteria’s pathogen inocula were cultured for the entire night at 37 °C. The antibiofilm test began by combining the fixed microorganisms in the test tubes with roughly 0.5 cc of the liquid nutrient broth, and then incubating them for the entire night at 37 °C. Second, following incubation, all tubes—treated and untreated—were disposed of. All tubes under investigation were then cleaned using phosphate buffer saline (PBS; pH 7.0) and, lastly, repeatedly rinsed with deionized water. Following that, the attached microbial cells in the tubes under investigation need to be fixed for approximately 15 min using 5.25 ml of 3.5% sodium acetate before being repeatedly cleansed with deionized water. The next step is to measure the semi-qualitative antibiofilm activity of the samples by staining the cleaned tubes containing the preserved microbial biofilm for approximately 15 min using 5 ml of 0.15% crystal violet (CV). Lastly, the CV-stained microbial cells were dissolved using ethanol solution (5 ml) to ascertain the samples’ semi-quantitative antibiofilm potential. The O.D. of the dissolved CV was then measured and counted following the application of the UV–vis. spectrophotometry method at a fixed wavelength (570 nm), and the microbial biofilm inhibition percentage was calculated using the following formula (El-Batal et al. 2019):

4 $$\begin{gathered} \% Biofilm\,~inhibition = {\left[ {{{\left( {O.D.\,control~\,sample - O.D.\,treated~\,sample} \right)}}}\right.}/\\ \quad\:{\left.{{O.D.\,control~\,sample}} \right]} \times 100 \end{gathered}$$

#### Anticancer activity of MPEE

To assess MPEE’s anticancer properties, Science Way Lab for Scientific Services in Cairo, Egypt, employed an MTT test. The cells were spined at 2500 rpm for 5 min at 4 °C in a microplate-compatible centrifuge (CAPPRondo Microplate Centrifuge, Germany) and then carefully aspirated from a 96-well plate in order to calibrate the MTT test. To ensure that every sample included the same quantity of the current media, 50 µl of serum-free medium and 50 µl of MTT solution were added to each well (Hanwell et al. 2012). They next incubated the dish for three hours at 37 °C. ATB-37 (Caco2) human colon epithelial cell lines were regularly cultivated in Roswell Park Memorial Institute (RPMI) media. Fetal bovine serum (FBS) was supplemented with 10% Penicillin G sodium, streptomycin sulfate, 250 g/ml Amphotericin B, and 2 mM L-glutamine. When sub-confluent, keep cells in humidified air with 5% CO_2_ at 37 °C. Monolayer cells were then gathered for subculturing after trypsin/EDTA treatment was administered at 37 °C. Cells were then used after 75% confluence was achieved. To treat the cells, a second aliquot of 100 µl of media containing various medication dosages became available. Following 48 h of drug exposure, the medium was disposed of, and 100 μl of phosphate buffer solution (PBS) in each well was mixed with 20 μl of 1 mg/ml stock solution. The mixture was then incubated for 4 h at 37 °C. After then, 100 μl of pure DMSO was used to dissolve the formazan crystals that had formed. We used an ELISA plate reader (FLUOstar OPTIMA, BMG LABTECH GmbH, Ortenberg, Germany) to quantify the absorbance of formazan solutions at λmax 570 nm.

### Molecular docking experiment

#### Protein preparation

The UniProt database provided the protein structures for the following organisms: NFKB: Q63369 and COX2: P00406. Using AutoDock Tools 1.5.7 (Liu 2022), the protein structures were ready for docking tests.

#### Ligand preparation

Phytochemicals were retrieved from the PubChem database (Trott and Olson 2010). The MMFF94 force field in Avogadro 1.2.0 was used to decrease the compounds’ energy because of their organic origin (BIOVIA 2023). Using the CB-Dock 2 service, the binding sites for the proteins were predicted (Quintana et al. 2021).

#### Molecular docking

AutoDock Vina was used to carry out the molecular docking. Using BIOVIA software, the docked conformations were examined and shown (Ribeiro et al. 2021).

#### Analysis of statistics

With the aid of IBM® SPSS® Statistics software (2017), all gathered data underwent statistical analysis. Applying a Duncan test with a *P*-value of 0.05 (Duncan 1955).

## Results

### Phytochemical screening of various MPEs

The presence of alkaloids, tannins, phenolics, flavonoids, steroids, and terpenoids in the fractions of mango peels was determined by phytochemical screening of four freshly made MPEs. The phytochemical profile showed that the different extracts are rich in a range of vital phytochemicals, as shown in Table 1. The phytoconstituent profiles of ethanolic and ethyl acetate extracts are identical, and the ethanolic extract produced three negative responses in tests for terpenoids, alkaloids, and steroids. It also yielded a negative result in the saponin test. The findings of a qualitative phytochemical screening of a chloroform extract of mango peels showed that steroids, terpenoids, and alkaloids were present, but glycosides, tannins, saponins, and polyphenols were not. However, as Table 1 shows, butanol extract contains phytochemicals like phenolic compounds, flavonoids, and coumarin.

**Table 1:** Phytochemical screening of various mango peels extracts (MPEs)

Phytochemical constituents	Ethyl acetate extract	Ethanol extract	Chloroform extract	Butanol extract
Alkaloids	+	−	+	−
Tannins	+	+	−	−
Flavonoids	+ +	+ +	−	+
Steroids	+ +	−	+ +	−
Saponins	−	−	−	−
Phenolic compounds	+ + +	+ + +	−	+ +
Glycosides	+ +	+	−	−
Coumarin	+ +	++	−	+
Terpenoids	+ +	−	+ + +	−

### Antioxidant activity and antioxidant agents (phenols, flavonoids and vitamin C) contents in MPEE

**Fig. 1:**
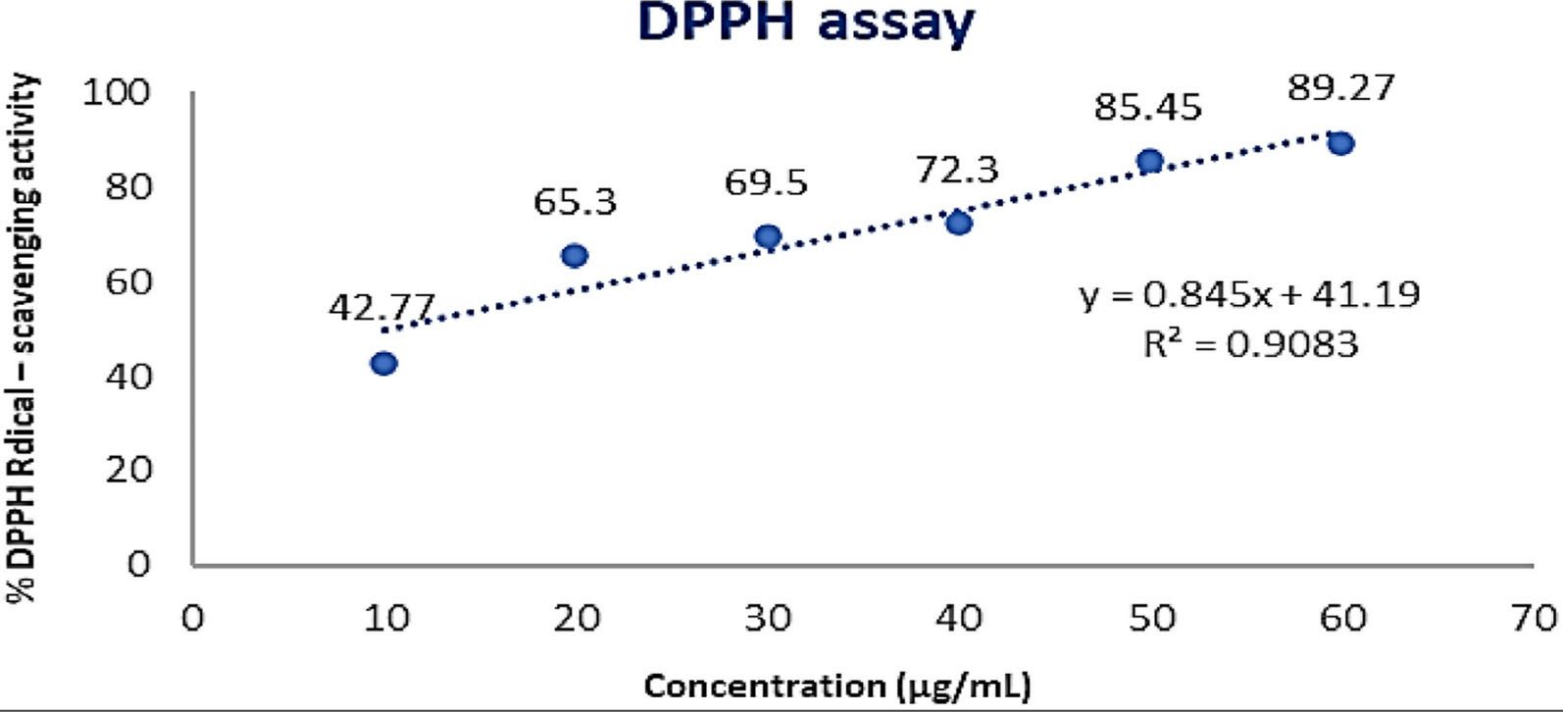
Antioxidant activity assessment of MPEE

According to the examination of the biologically active components of MPEs in Table 1 which note that the MPEE was rich in compounds that have high activity as antioxidants and antimicrobial. Therefore, compounds that have an effect as antioxidants, such as phenols, flavonoids and vitamin C, were estimated in ethyl acetate extract. The antioxidative potential of mango peels must be retained for the observed high phenolic compounds content (4110.9 ± 2.06 μg/g DW), flavonoids content (756.09 ± 1.50 μg/g DW) and vitamin C (0.28 ± 1.03%). The antioxidant capacity was evaluated by testing the phenolic and flavonoid contents of MPEE using a DPPH radical scavenging capacity assay. MPEE exhibited the highest percentage of radical scavenging activity (89.27%) at 60 μg/ml, followed by 50 μg/ml (85.45%), based on the DPPH assay (Fig. 1). Evidently, the antioxidant activity rose in tandem with the MPEE concentration, presumably as a result of the extract’s active ingredients being more concentrated. The IC_50_ value, or the concentration of the sample required to scavenge 50% of DPPH, demonstrated antioxidant activity. The DPPH assay yielded an IC_50_ value of 10.426 μg/ml.

### GC–MS analysis of MPEE

**Fig. 2:**
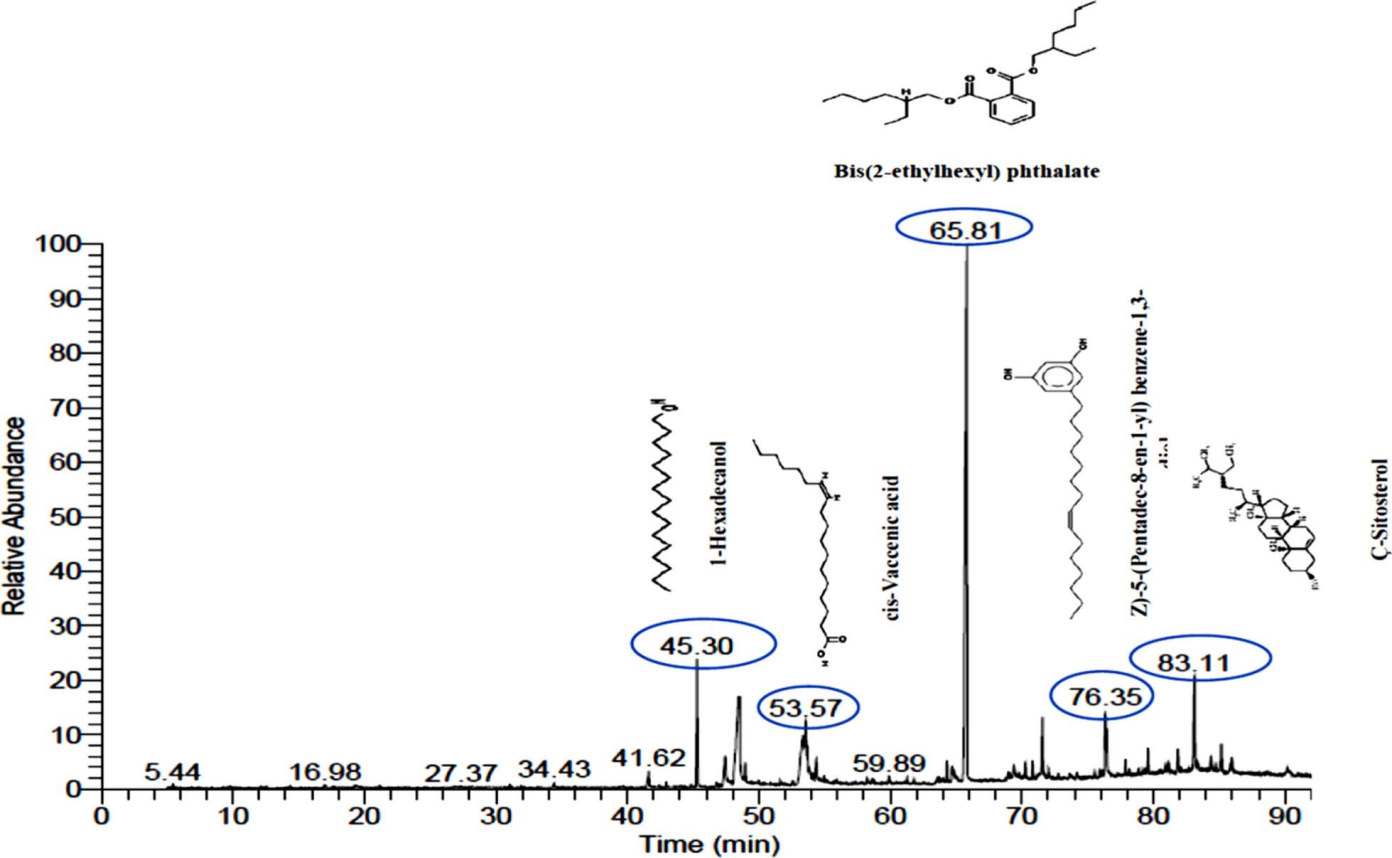
GC–MS spectra of MPEE and the chemical structure of some of the constituents

Using the MPEE’s retention duration, experimental m/z, MS/MS fragments, database differences (library), metabolite class, and suggested compounds, the phytoconstituents were identified using GC–MS. The GC–MS chromatogram for the ethyl acetate extract is shown in Fig. 2. The GC–MS analysis of the mango peel ethyl acetate extract revealed 33 potential phytochemical components, which are shown in Table S2. The primary components of MPEE are bis(2-ethylhexyl) phthalate (37.67%), 1-hexadecanol (5.09%), and n-hexadecenoic acid (6.41%). Using GC–MS analysis, the amounts of vitamin E (1.00%), Ç-Sitosterol (4.25%), cis-vaccenic acid (3.56%), and l-(+)-ascorbic acid 2,6-dihexadecanoate (0.12%) contents were determined in order to better concentrate on the compounds with very effective antioxidative and antibacterial activity.

### Quantitative analysis of MPEE polyphenolic compounds identified by HPLC

**Fig. 3:**
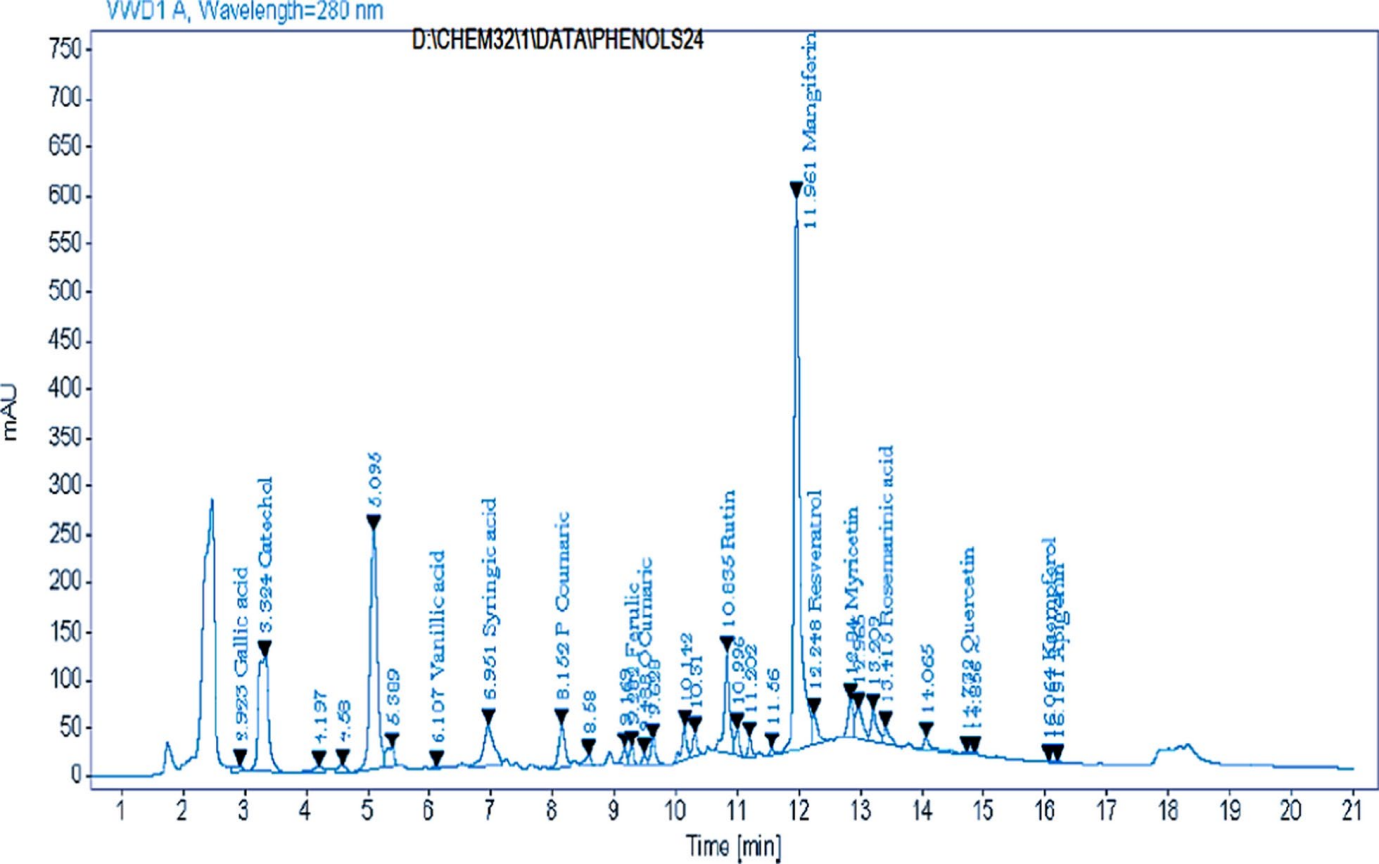
HPLC chromatogram of *M. indica* L. peels ethyl acetate extract (MPEE)

Using HPLC, a phytochemical examination of MPEE revealed the presence of nine phenolic compounds: resveratrol, gallic acid, catechol, vanillic acid, syringic acid, p-coumaric acids, ferulic acid, o-coumaric acid, and rosemarinic acid (see Fig. 3 and Table S3). Also, we found in MPEE four flavonoids (myricetin, quercetin, kaempferol and apigenin) and two glycosides (rutin and mangiferin).

### Anti-foodborne pathogenic activity investigation of MPEE

**Fig. 4:**
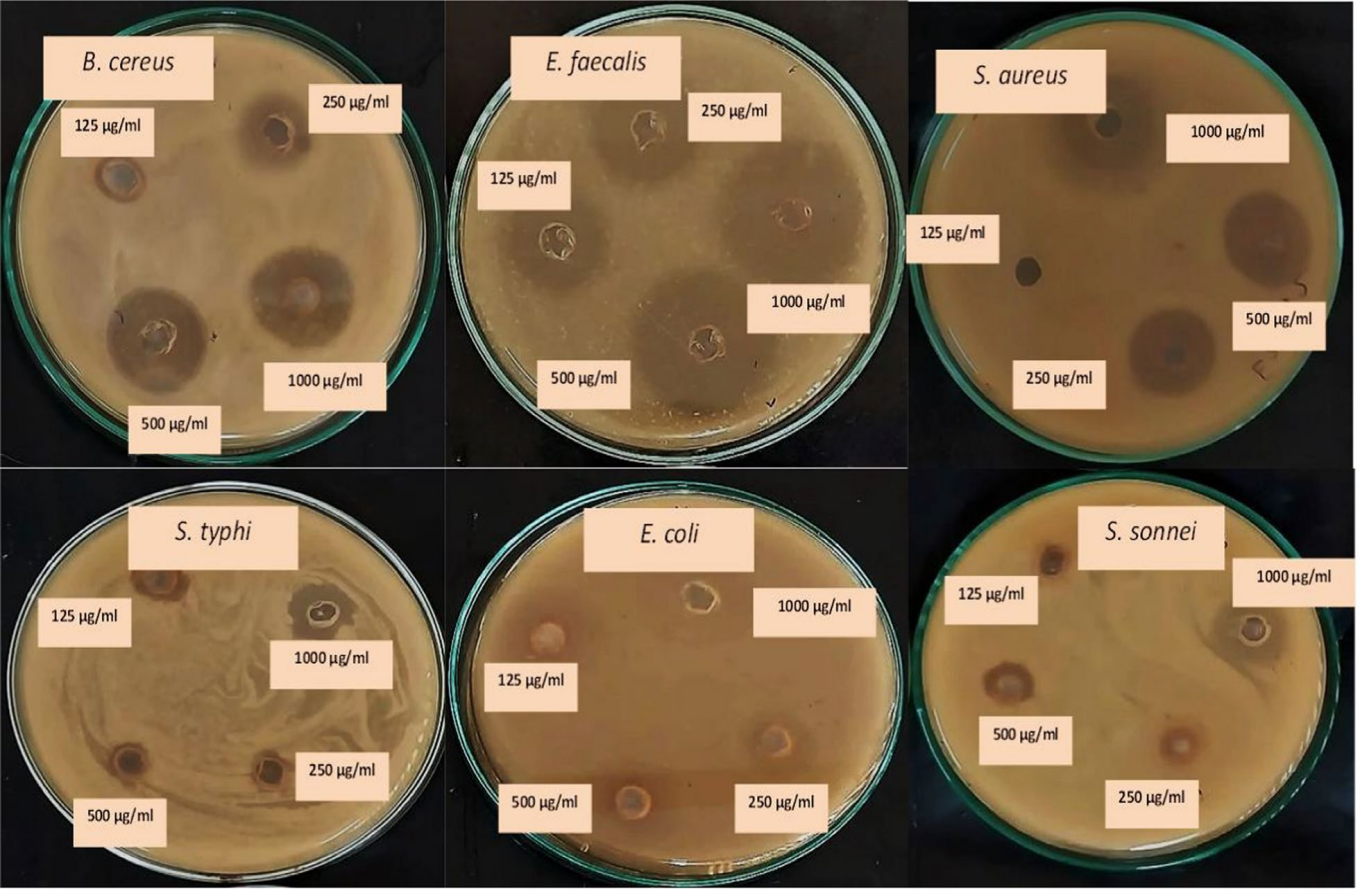
Anti-food borne pathogen bacteria activity of different concentrations of MPEE using well agar diffusion method after incubation at 37 °C for 24 h

According to the data in Table 2 and Fig. 4, MPEE caused IZD on well-agar diffusion plates that ranged from 10.0 to 32.0 mm for all tested pathogenic bacteria, while the control antibiotic caused IZD that ranged from 30 to 40 mm. Both *B. cereus* and *E. faecalis* had the most significant IZD, measuring 32.0 and 30.0 mm, respectively, and having activity index (AI) values of 0.91 and 1.0. *E. coli* and *S. sonnei*, on the other hand, had the lowest IZDs, measuring 15.0 and 12.0 mm, respectively, and 0.45 and 0.34 of AI.

**Table 2:** Food-borne pathogenic bacterial strains’ inhibition zone diameter (IZD) affected by MPEE in compared to a control antibiotic following a 24 h incubation period at 37 °C

Parameters	Food borne pathogen strains					
MPEE (μg/ml)	G + ve strains			G-ve strains		
	*E. faecalis *ATCC 7080	*B. cereus* ATCC 11778	*S. aureus *ATCC 5638	*E. coli* ATCC 8739	*S. sonnei* DSM 5570	*S. typhi* DSM 17058
1000	32.0^c^ ± 0.43	30.0^d^ ± 0.53	21.0^h^ ± 0.11	15.0^k^ ± 0.39	12.0^l^ ± 0.42	20.0^h^ ± 0.36
500	28.0^e^ ± 0.40	28.0^e^ ± 0.04	20.0^h^ ± 0.17	12.0^l^ ± 0.01	10.0^m^ ± 0.65	19.0^i^ ± 0sss.58
250	16.0^j^ ± 0.11	25.0^f^ ± 0.40	20.0^h^ ± 0.04	10.0^m^ ± 0.11	10.0^m^ ± 0.77	10.0^m^ ± 0.35
125	0.00	21.0^h^ ± 0.33	18.0^i^ ± 0.08	0.00	10.0^m^ ± 0.21	0.00
Standard antibiotics (1000 μg/ml)	Streptomycin			Ampicillin		
	35.0^b^ ± 0.21	30.0^d^ ± 0.33	40.0^a^ ± 0.20	33.0^c^ ± 0.37	35.0^b^ ± 0.29	40.0^a^ ± 0.25
AI	0.91	1.0	0.53	0.45	0.34	0.53

### Evaluation of MIC and MBC of MPEE

The MIC values of MPEE against the tested food-borne pathogenic bacteria ranged from 250 to 1000 μg/ml, according to the results in Table 3. The MIC value for all tested food-borne pathogen bacteria was 250 μg/ml MPEE. Except, *E. coli* showed a 500 μg/ml MIC value. At concentrations between 500 and 1000 μg/ml, the data clearly demonstrate 100% of MPEE’s antibacterial spectrum activity, in contrary at dose of 250 μg/ml, the activity was assessed to be 83.3%. Furthermore, the examined pathogen strains showed no antibacterial action at a dosage of 125 μg/ml. Table 4 shows that the MBC values of MPEE against the investigated food-borne pathogenic bacterial strains ranged from 500 to 1000 μg/ml. The MBC values for *B. cereus*, *E. faecalis*, and *S. typhi* were 500 μg/ml MPEE. On the other hand, the MBC value for *S. aureus*, *S. sonnei*, and *E. coli* was 1000 μg/ml. At 1000 μg/ml, the data clearly demonstrate 100% of MPEE’s antibacterial spectrum activity, while at 500 μg/ml, the activity was 50.0%. However, none of the investigated food pathogen strains showed antibacterial influence at doses between 125 and 250 μg/ml.

**Table 3:** The minimal inhibitory concentration (MIC) and spectrum activity of MPEE against food-borne pathogenic pathogens following a 24 h incubation period at 37 °C

Parameters	Food borne pathogen strains							
MPEE(μg/ml)	G + ve strains			G-ve strains			Spectrum Activity (%)	
	*E. faecalis* ATCC 7080	*B. cereus* ATCC 11778	*S. aureus* ATCC 5638	*E. coli* ATCC 8739	*S. sonnei* DSM 5570	*S. typhi* DSM 17058		
1000	−	−	−	−	−	−	6/6	100
500	−	−	−	−	−	−	6/6	100
250	−	−	−	+	−	−	5/6	83.3
125	+	+	+	+	+	+	0/6	0.00
MIC score (μg/ml)	250	250	250	500	250	250

**Table 4:** MPEE’s minimum bactericidal concentration (MBC) and spectrum activity against food-borne pathogenic pathogens following a 24-h incubation period at 37 °C

Parameters	Food borne pathogen strains							
MPEE (μg/ml)	G + ve strains			G-ve strains			Spectrum Activity (%)	
	*E. faecalis* ATCC 7080	*B. cereus* ATCC 11778	*S. aureus* ATCC 5638	*E. coli* ATCC 8739	*S. sonnei* DSM 5570	*S. typhi* DSM 17058		
1000	−	−	−	−	−	−	6/6	100
500	−	−	+	+	+	−	3/6	50.0
250	+	+	+	+	+	+	0/6	0.00
125	+	+	+	+	+	+	0/6	0.00
MBC score (μg/ml)	500	500	1000	1000	1000	500		
MBC/MIC ratio	2	2	4	2	4	2		
Impact	Bactericidal	Bactericidal	Bacteriostatic	Bactericidal	Bacteriostatic	Bactericidal		

### Antibiofilm activity of MPEE

**Fig. 5:**
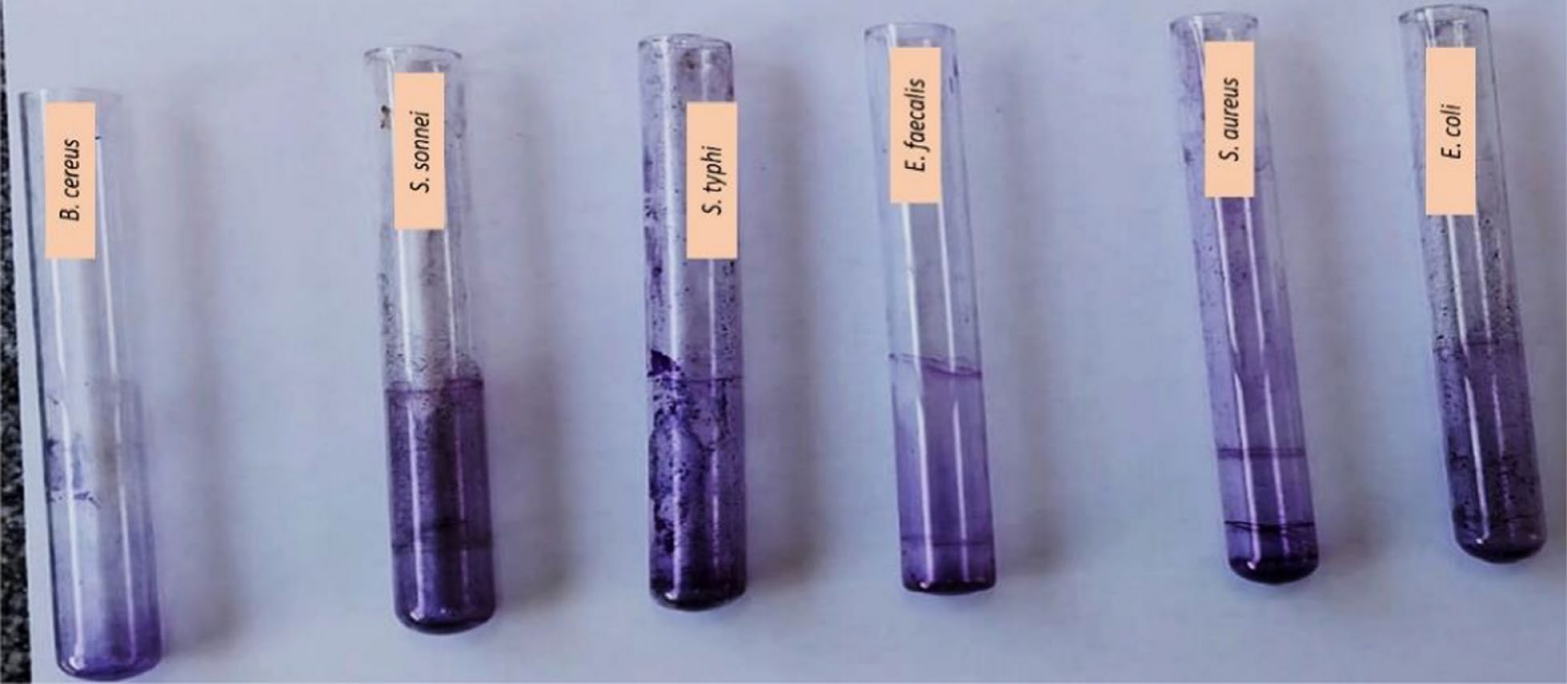
Antibiofilm activity of food borne pathogen bacteria treated with 1000 μg/ml MPEE by tube method

The MPEE percentages of biofilm inhibition were represented in Table S4 and Fig. 5. The biofilm inhibition percentage of MPEE was as follows: *B. cereus* (98.75%) ≥ *E. faecalis* (96.38%) ≥ *S. aureus* (90.45%) ≥ *S. typhi* (85.92%) ≥ *E. coli* (62.96%) ≥ *S. sonnei* (53.33%). So, it could be observed that gram positive (G + ve) strains represented the most influenced strains compared to the gram negative (G-ve) strains.

### Anticancer activity of MPEE

**Fig. 6:**
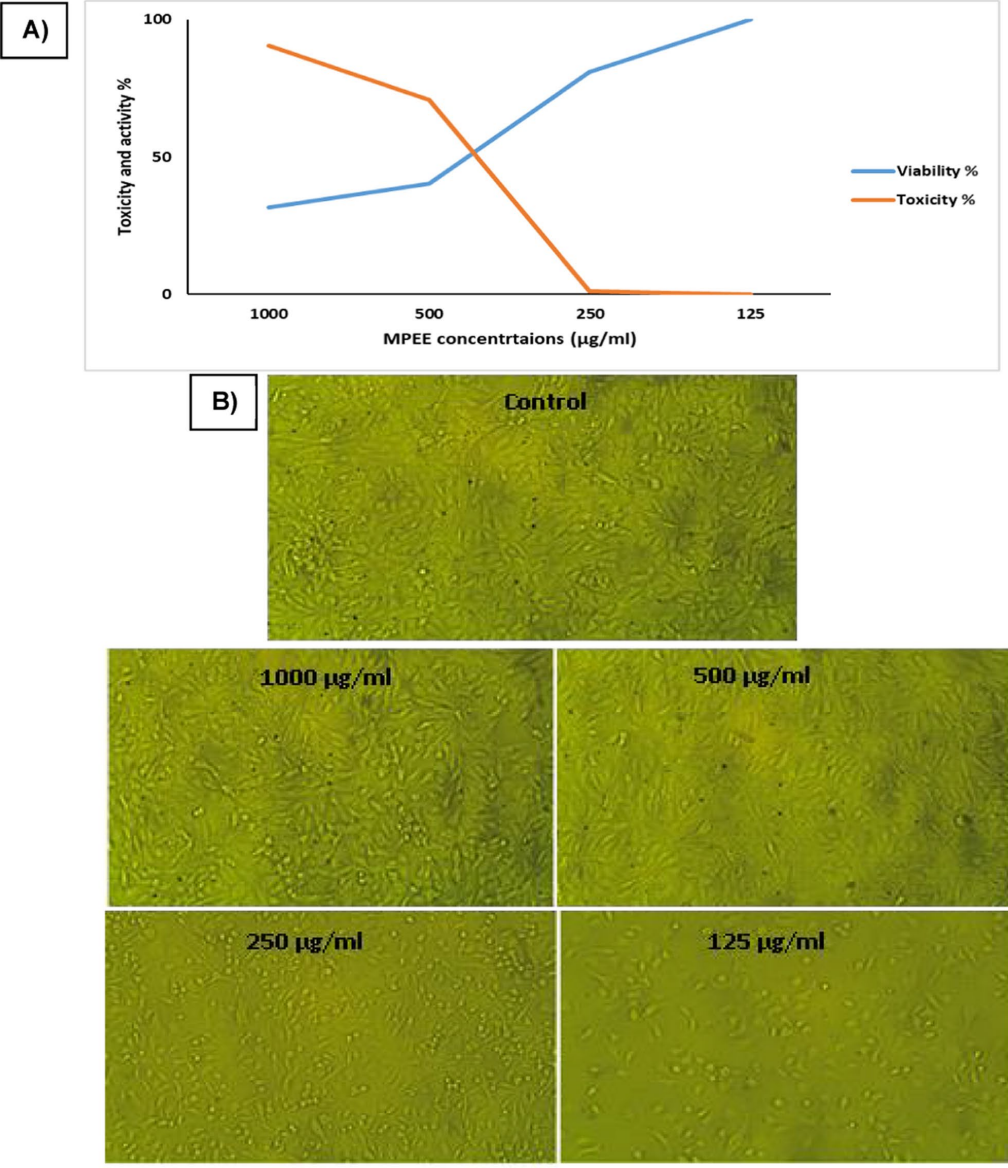
**A** Dose dependent curve and IC_50_ of different concentrations of MPEE on human colon epithelial ATB-37 carcinoma (Caco2). **B** Different MPPE concentrations have an impact on cell viability examined by microscopic images for human colon epithelial carcinoma ATB-37 (Caco2)

It could be observed from Fig. 6A and B that the anticancer effect of MPEE on Human colon epithelial ATB-37 (Caco2) using different doses ranged between 1000 and 125 µg/ml was as follows: 90.39, 70.76, 1.04, 0.00% when using 1000, 500, 250, and 125 µg/ml, respectively with cell viability of 31.60, 40.23, 80.955, and 100% in same arrangement. The IC_50_ dose was calculated and found to be at 430.36 µg/ml of MPEE. Images taken with a light microscope demonstrating the antiproliferative action of MPEE against human colon epithelium ATB-37 (Caco2) revealed extensive morphological alterations that take place at the cell surface or in the cytoskeleton and can be tracked and linked to cell survival. Large volume reductions brought on by protein and intracellular ion losses as a result of altered permeability to sodium or potassium are another way to spot damage. Necrotic cell characteristics also include nuclear enlargement, chromatin flocculation, and absence of nuclear basophilia. In apoptotic cells, nuclear condensation, nuclear fragmentation, and cell shrinkage occur.

### Molecular docking major active compounds in MPPE with two target colon proteins (COX2 and NFKB)

Table S5’s docking data provide information about the different drugs’ binding affinities against the COX2 and NFKB proteins. Lower ΔG values signify stronger binding and heightened inhibitory potential. Rutin emerges as a noteworthy compound, displaying robust binding affinity for both targets. Particularly, rutin exhibits the strongest binding affinity for COX2 among the tested compounds, with a ΔG value of − 5.5 kcal/mol. For NFKB, rutin, along with several other compounds including rosemarinic acid, resveratrol, myricetin, and quercetin, showcases substantial binding affinities, with rutin leading with a ΔG value of − 7.6 kcal/mol. The concurrent promising binding affinities of rutin, apigenin, kaempferol, mangiferin, myricetin, quercetin, and resveratrol for both COX2 and NFKB hint at their potential inhibitory activity against these crucial targets in inflammation pathways.

Turning to the specific interactions of rutin with NFKB, Figure S1 and Table S6 elucidate its binding mode. Rutin engages in diverse interactions including conventional hydrogen bonding with ALA283:O, and hydrophobic interactions such as pi-pi stacking with TYR340, amide-pi stacking with ALA287 and ALA288, and pi-alkyl interactions with multiple residues including LEU284, ALA287, and ALA288. These interactions, as highlighted by their distances, collectively contribute to the strong binding affinity observed between rutin and NFKB.

Similarly, the interactions of rutin with COX2, depicted in Figure S2 and detailed in Table S7, underscore its binding mechanisms. Rutin forms hydrophobic interactions, have pi-pi stacked interactions with TRP65 and pi-alkyl interactions with PRO69. Notably, the stability of the binding mode is supported by hydrophobic interactions with TRP65 occurring at varying distances, indicative of a robust binding configuration.

In addition to that, the interactions of mangiferin, a major compound in MPEE with NFKB, depicted in Figure S3A and detailed in Table S8, underscore its binding mechanisms. Mangiferin forms hydrophobic interactions, including pi-alkyl interactions with ALA283 and ALA287 and pi-Sigma and pi-pi stacking interactions with TYR340. On the other hand, the interactions of mangiferin with COX2 are shown in Figure S3B and Table S8. With TRP65, mangiferin produces hydrophobic interactions, such as pi-pi stacking interactions.

As shown in Figure S4A and Table S9, there were hydrophobic interactions between Bis(2-ethylhexyl) phthalate with NFKB, including Pi-Alkyl stacked interactions with LEU211 and Alkyl interactions with LEU253, LEU388 and LEU391. Also, Bis(2-ethylhexyl) phthalate forms Hydrogen Bond with GLN215. The interactions of Bis(2-ethylhexyl) phthalate with COX2 are shown in Figure S4B and Table S9. Pi-Sigma stacked contacts with TRP65 and Alkyl stacked interactions with LEU68 and PRO69 are two examples of the hydrophobic interactions that bis(2-ethylhexyl)phthalate can create. Pi-Alkyl stacking interactions with TRP65 are also present.

## Discussion

Mango peels, rich in antibacterial and antioxidant substances, can be used to create new products, reduce bio-waste, and protect against food-borne pathogens and colon cancer. Limited research explores their potential as phytochemical entities and antimicrobial therapy (Mwaurah et al. 2020; Zhanpeng et al. 2024; Ala˜n´on et al. 2021). The study reveals mango peel extract as a potential food-borne pathogen treatment, with MPEE being the most effective due to its efficient extraction of polar and non-polar compounds. MPEE contains vitamin E, steroids, terpenoids, and unsaturated fatty acids (Coelho et al. 2019; Lebaka et al. 2021; Kabir et al. 2024). Mango peels, rich in antioxidant and anti-tumor properties, are used in functional and nutraceutical products. Factors like genome, environmental conditions, harvest stage, extraction techniques, and solvent polarity influence these properties (Martínez-Ramos et al. 2020; Sarkar et al. 2022; Tariq et al. 2023; Suttisansanee et al. 2024).

The antioxidant activity of mango extracts is attributable to the total antioxidant capacity of the constituent phenolic compounds or to certain components that exhibit substantial antioxidant capacity even at low concentrations (Khennouf et al. 2003; Barchan et al. 2014). Other factors include solvent nature, extraction technique, time, temperature, sample/solvent ratio, and sample composition. Ethanol is preferred due to its efficiency (Zekri et al. 2021; Zahid et al. 2022; García-Mahecha et al. 2023).

Zekri et al. (2021) discovered that the extracts of *M. pulegium* ethyl acetate and methanol had the highest concentrations of polyphenols, respectively. The solvents used in extraction affect hydrophilic phenols, while chloroform extracts had low polyphenol levels. Furthermore, our results showed that the ethyl acetate extract from mango peels had the highest extraction efficiency for both hydrophilic and hydrophobic components. Bencheikh et al*.* (2012) found *Algerian pennyroyal* ethyl acetate extract and mango leaf EtOAC extracts have higher flavonoid content and phenolic and total flavonoids compared to MeOH extract. The MPEE identified fifteen compounds from three families (phenolic acids, flavonoids, and glycosides) in terms of phenolic profiles, which aligns with previous findings (García-Mahecha et al. 2023).

Variety, harvest stage, extraction method, solvent, and environmental factors all affect a fruit’s phenolic content. Mangiferin is the major phenolic compound in MPEE, followed by Catechol (Zahid et al. 2022). The antioxidant capacity of plant phenolics is a synergistic effect of all compounds, and MPE was found to be more effective against oxidative stress (Tacias-Pascacio et al. 2022). Their special functional groups and chemical structure make them effective antioxidants. Phenols can neutralize free radicals by contributing electrons or hydrogen (Nirmal et al. 2022).

When Ajila et al. (2007) examined the antioxidant capacities of many Indian mango peel varieties, they discovered that antioxidant activity was concentration-dependent and that the IC_50_ values of GAE ranged from 1.83 to 4.54 μg/ml. Mango by-products may contain natural phenolic antioxidants, fatty acids, sesquiterpenoids, phytosterols, and tocopherols, contributing to their potential health benefits (Dorta et al. 2014). MPEE exhibited high antioxidant capacity, scavenging DPPH radical due to mango peel’s high lipophilic antioxidants, phenolic compounds, mangiferin, Catechol, and rutin in the line with (Sumaya-Martínez et al. 2013).

Because the waste extracts from mango peels contained phytochemicals including mangiferin and other polyphenols, they demonstrated exceptional antioxidant and antibacterial properties (Koirala et al. 2024). The main component of all the by-products, mangiferin, demonstrated improved FRAP, DPPH radical scavenging, and ORAC capabilities (Kaur et al. 2022). Much research has been applied to mango by-products as a promising source of antioxidant-potential polyphenolic chemicals (Alaiya et al*.*^2023^). Phenolic chemicals, exemplified by gallic acid, gallotannins, ellagic acid, and catechin, have been found in the peel, pulp, and seed of Keitt, Ataulfo, Kent, and other varieties (Adamcyzyk et al. 2017).

It has also been demonstrated that mango by-products contain xantones, such mangiferin and its derivatives, which are particularly intriguing because of their bioactive properties, and flavonoids, which are represented by quercetin derivatives (Gutiérrez-Grijalva et al. 2020). The ethanolic extract of peels from mango had the lowest concentration of various chemicals, especially non-polar compounds like terpenes and compound steroids that are important in breaking down the walls of microbes and therefore have an impact on antimicrobial drugs. The ethyl acetate extract, on the other hand, showed the appearance of every phytochemical component examined.

MPEE significantly impacts G + ve food-borne pathogens, with *E. faecalis* and *B. cereus* having the highest rank compared to standard antibiotics against *S. aureus*, *S. sonnei*, *E. coli*, and *S. typhi*. Because of the closely packed peptidoglycans that make up *M. indica* and its peel, researchers have examined the antibacterial qualities of alkaloids, tannins, flavonoids, mangiferin, and phenolics (Posseti et al. 2020). The extraction method and solvent utilized determine the antibacterial qualities of mango extracts, which may also contain tannins or plant polyphenols (Shang et al. 2021).

In the study of Sánchez-Camargo et al. (2021) found gallotannic, a hydrolysable tannin extracted from mango kernel extract, has a strong antibacterial effect on *B. subtilis*. By creating an unstable combination between Fe^2+^ and Fe^3+^, it controls iron homeostasis.

Tannins, such as gallotannic, have antibacterial properties because they have a strong affinity for iron and may precipitate protein from bacteria. And found that G + ve bacteria are more susceptible to gallotannic than G-ve bacteria due to their charged outer membrane potentially limiting electrostatic repulsion. Alkaloids, a type of phytochemical with a basic nitrogen atom, form hydrogen bonds with enzymes, proteins, and receptors, gain antibacterial properties through mechanisms like efflux pump inhibition and nucleic acid synthesis inhibition (Timsina et al*.*^2015^). Also, Mango peel and ethanol extracts show strong antimicrobial activity, with 95% inhibition percentage for tested pathogens, *S. aureus* and MRSA are targets of the antibacterial action of mango kernel extracts (Percival et al. 2006).

Posseti et al. (2020) studied the inhibition zones, MIC, and MBC of three mango types against *E. coli* and *S. aureus*. According to the findings, the extracts of Coquinho mango peels, Coquinho mango kernels, Tommy Atkins mango peels, Tommy Atkins mango kernels, Espada mango peels, and Espada mango kernels exhibited the strongest bactericidal activity against *S. aureus* and *E. coli*. Whereas Ogidi et al. (2021) study found antimicrobial activity of *M. indica* stem-bark extracts against *Shigella* sp*.*, *E. coli*, *Staphylococcus* sp*.*, and *Vibrio* sp., with methanol extract showing highest IZD for *Staphylococcus* sp.

The antibiofilm inhibition percentage of MPEE was assessed using UV–vis spectroscopy at 570 nm. Results showed negative results for *B. cereus* and *E. faecalis*, with a creamy-yellow surface matt attached to test tubes. Positive results showed either no CV color or a slight violet tint. Antimicrobial activity, physicochemical properties, penetrating capabilities, and charge connection are some of the factors that affect inhibitory percentage (du Plessis-Stoman et al. 2011). It was noticed that the antibiofilm activity might be due to controlling the exopolysaccharide secretion (Shoji et al. 2011).

The MPPE showed antitumor effects against human colon cancer cells, while mango peels and flesh showed antiproliferative activities in various cancer lines, as confirmed by various studies (Daud et al. 2010; Amr et al. 2023). Mango pulp, peel, and kernel extracts show anticancer effects, with significant antiproliferative effects against HeLa cells, breast, colon, renal, and K562 leukemia cells, as confirmed by (Trease and Evans 2021; Farouk et al. 2023). Mangiferin and tannins in mango juice and extracts show anticancer activity against HL-60 cells, potentially disrupting cell adhesion and attachment, inhibiting telomerase and genes, and enhancing cellular apoptosis (Daud et al. 2010; Amr et al. 2023).

The docking analysis of mango peel ethyl acetate extract reveals rutin, bis(2-ethylhexyl) phthalate, and mangiferin as potential inhibitors of COX2 and NFKB proteins, offering potential for further research. As indicated by (Gökbulut 2015) inflammation, diabetes, immunomodulation, and tumor prevention are among the human cancer cell lines that have been shown to be susceptible to the anti-proliferative effects of mangiferin, a bioactive xanthone glucoside found in mango peels. It also stimulates endothelial cells, potentially treating vascular disorders. The study reveals that mango peel ethyl acetate extract contains numerous active biological compounds, including phenolic acids, flavonoids, vitamins C and E, steroid and terpenoids, which contribute to its antioxidant properties and antibacterial activity against pathogenic bacterial strains, thus inhibiting various generative diseases like food-borne pathogens and colon cancer.

Finally, according to this study, MPEE includes a variety of phytochemicals, including terpenoids, polyphenols, carotenoids, and tocopherols. In addition, agricultural waste was reused to produce and extract compounds of therapeutic importance for colon cancer and protection from pathogenic bacteria transmitted through food from mango peels. It can also reduce the environmental impacts resulting from mismanagement of the disposal of these wastes. In general, ethyl acetate solvent can generate a greater yield of antioxidant agents than other solvents of mango peels are under the same range extraction parameters. as well as phytochemical screening, HPLC and GC–MS analysis, and antimicrobial activity of MPEE the results obtained from mango peels were relatively better than other mango peel extracts. In conclusion, based on the experimental results, MPEE has antioxidant, antimicrobial, and colonic antitumor potential to replace other expensive sources.

## Supplementary Information

Additional file1 (PDF 682 KB)

## Data Availability

This study didn’t contain associated data.
