# Supplementary material for: In vitro investigation of Mangifera indica L. peel extracts: antibacterial, antioxidant, and docking studies
Source: AMB Express. 2025 May 8;15:73. doi: 10.1186/s13568-025-01882-w (PMC12061830; doi:10.1186/s13568-025-01882-w)
Supplement: Supplementary file 1 — Additional file1 (PDF 682 KB) [file 13568_2025_1882_MOESM1_ESM.pdf]

Supplementary file

Supplementary Tables ligands

Table S1 Experimental phytochemical tests on various Mango peels extracts (MPE).
Table S2 Bioactive compounds identified in the ethyl acetate extract of *M. indica* L. peels by GC-MS.
Table S3 Quantitative analysis of some phytochemicals identified by HPLC in MPEE.
Table S4 Biofilm formation inhibition % for treated and non-treated food borne pathogenic bacteria with 1000 µg/ml MPEE.
Table S5 ΔG binding affinity (Kcal/mol) for active polyphenol compounds extracted from MPPE with COX2 and NFKB.
Table S6 Interactions between Rutin (best docked polyphenol compound) with NFKB.
Table S7 Interaction between Rutin and COX2.
Table S8 Interactions between Mangiferin with NFKB and COX2.
Table S9 Interactions between Bis(2-ethylhexyl) phthalate with NFKB and COX2.

Table S1

Phytoconstituents	Tests for phytochemical components	Observation
Glycosides	1 ml extract + 2 drops of lead acetate solution + filter + 2 ml of chloroform + 2 drops of glacial acetic acid + $FeCl_3$ solution + 2 ml conc H_2SO_4	A brown ring at the interface and green color in the acetic layer were observed
Alkaloids	1 ml extract + 2 drops of Dragendorff's reagents	The resulting solution was not turbid
Saponins	1 ml of extract + 10 ml of distilled H_2O in 2.5 ml filtrate + 2 drops of olive oil + vigorous shaking for 2 min	Persistent foam was observed
Tannins	1 ml of extract + 5 ml of distilled H_2O + 2 drops of 10% $FeCl_3$ solution	A brownish green precipitate indicated presence of tannins
Flavonoids	1 ml extract + 1 ml $Pb(C_2H_3O_2)_4$ (10%)	Yellow coloration
Coumarins	2 ml extract + 3 ml NaOH (10%)	Yellow coloration
Phenolic compounds	1 ml of extract + 5 ml of distilled H_2O + 2 drops of 10% $FeCl_3$ solution	Brownish-yellow coloration was observed
Steroids	2 ml extract + 2 ml $CHCl_3$ + 2 ml H_2SO_4 (conc.)	Formation of the bilayer (red top layer and greenish bottom layer)
Terpenoids	2 ml extract + 2 ml $CHCl_3$ + 2 ml H_2SO_4 (conc.) heat for 2 min	A reddish-brown color

Table S2

No.	Compound Name	Molecular Formula	Chemical structure	RT (min)	Area%
1	Octadecanoic acid, 2,3-dihydroxypropyl ester	$C_{21}H_{42}O_4$	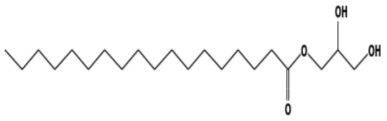	9.21	0.07
2	3,4-Dimethoxycinnamic acid	$C_{11}H_{12}O_4$	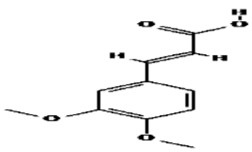	12.49	0.07
3	Nonanal	$C_9H_{18}O$	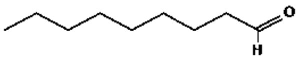	14.38	0.07
4	2-Decenal, (E)-	$C_{10}H_{18}O$	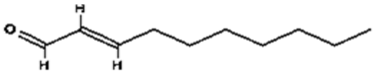	21.17	0.08
5	2(4H)-Benzofuranone, 5,6,7,7a-tetrahydro-4,4,7a-trimethyl-, (R)-	$C_{11}H_{16}O_2$	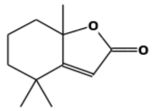	31.07	0.11
6	Dodecanoic acid	$C_{12}H_{24}O_2$	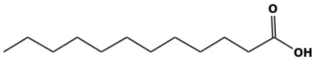	34.43	0.21
7	Tetradecanoic acid	$C_{14}H_{28}O_2$	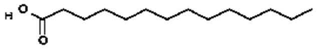	41.62	0.87
8	Octadecanoic acid, ethyl ester	$C_{20}H_{40}O_2$	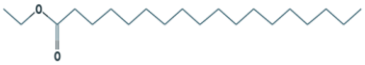	42.42	0.09
9	Isochiapin B	$C_{19}H_{22}O_6$	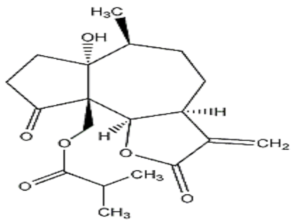	43.58	0.07

10	Ethanol, 2-(9-octadecenyloxy)-, (Z)-	$C_{20}H_{40}O_2$	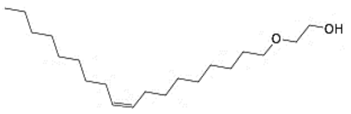	44.07	0.07
11	Pentadecanoic acid	$C_{15}H_{30}O_2$	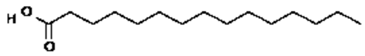	44.83	0.13
12	1-Hexadecanol	$C_{16}H_{34}O$	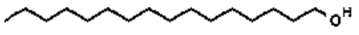	45.30	5.09
13	cis-11-Eicosenoic acid	$C_{20}H_{38}O_2$	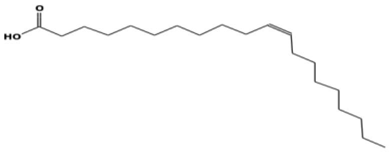	47.03	0.10
14	Palmitoleic acid	$C_{16}H_{30}O_2$	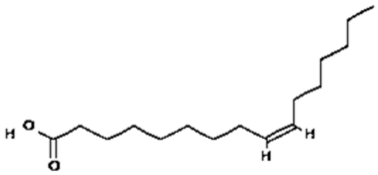	47.43	1.95
15	Ppropiolic acid, 3-(1-hydroxy-2-isopropyl-5-methylcyclohexyl)-	$C_{13}H_{20}O_3$	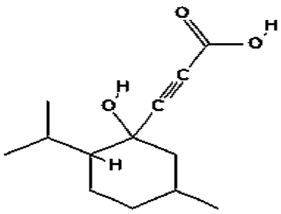	47.87	0.10
16	n-Hexadecanoic acid	$C_{16}H_{32}O_2$	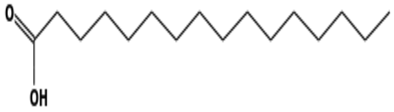	48.11	6.41
17	Hexadecanoic acid, ethyl ester	$C_{18}H_{36}O_2$	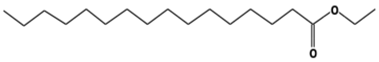	48.95	0.63

18	l-(+)-Ascorbic acid 2,6-dihexadecanoate	$C_{38}H_{68}O_8$	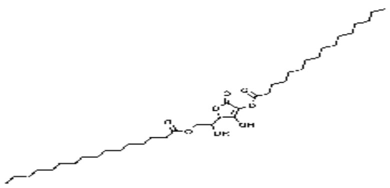	49.98	0.12
19	Hexadecanoic acid, 2,3 dihydroxypropyl ester	$C_{19}H_{38}O_4$	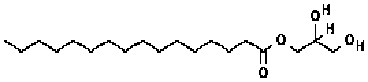	49.41	0.07
20	cis-Vaccenic acid	$C_{18}H_{34}O_2$	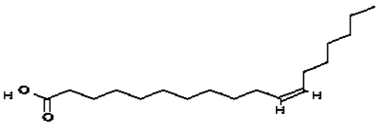	53.57	3.56
21	9,12,15-Octadecatrienoic acid,2,3,dihydroxypropyl ester, (Z,Z,Z)	$C_{19}H_{32}O_2$	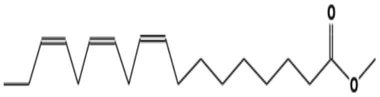	53.91	0.13
22	Oleic Acid	$C_{18}H_{34}O_2$	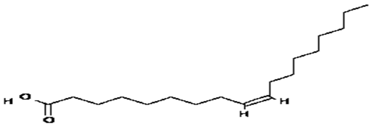	55.72	0.10
23	Phenol,2,2'-methylenebis[6-(1,1dimethylethyl)-4-methyl	$C_{23}H_{32}O_2$	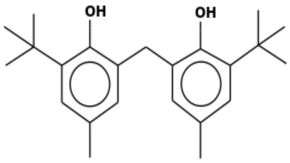	61.28	0.26
24	Bis(2-ethylhexyl) phthalate	$C_{24}H_{38}O_4$	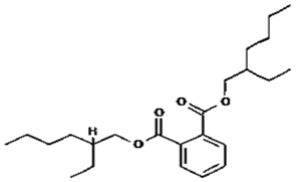	65.80	37.67

25	Rhamnetin-3-O-glucoside	$C_{22}H_{22}O_{11}$	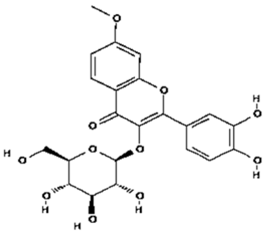	70.00	0.08
26	4H-1-BENZOPYRAN-4-ONE, 2-(3,4-DIHYDROXYPHENYL)-6,8-DI-á-D-GLUCO PYRANOSYL-5,7-DIHYDROXY-	$C_{21}H_{20}O_{12}$	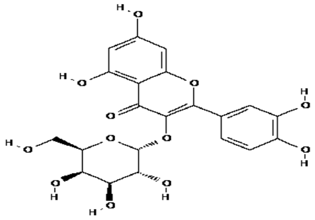	71.34	0.11
27	13-Docosenamide, (Z)-	$C_{22}H_{43}NO$	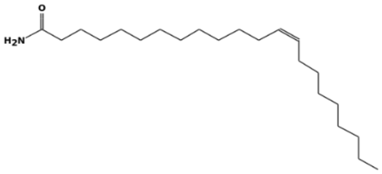	71.56	2.55
28	(Z)-5-(Pentadec-8-en-1-yl) benzene-1,3-diol	$C_{21}H_{34}O_2$	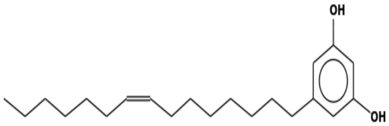	76.35	2.80
29	Morphinan-6α-ol, 7,8-didehydro-4,5α-epoxy-3-methoxy-17-methyl-, acetate (ester)	$C_{20}H_{23}NO_4$	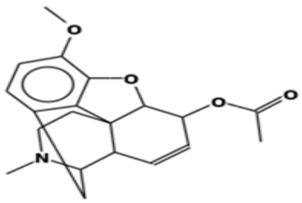	77.89	0.61
30	Vitamin E	$C_{30}H_{54}O_2$	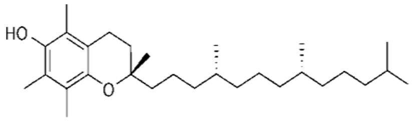	79.59	1.00

31	Ç-Sitosterol	$C_{29}H_{52}O$	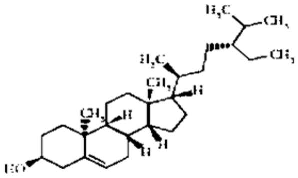	83.11	4.52
32	Lupeol	$C_{30}H_{50}O$	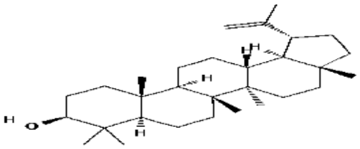	84.38	0.75
33	Hexadecanoic acid, hexadecyl ester	$C_{32}H_{64}O_2$	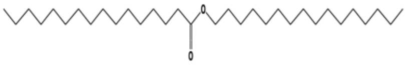	85.17	1.43

Table S3

No.	Compound	RT (min)	Area	Concentration (mg/kg)
		**Phenolic constituents**		
1	Gallic acid	2.92	48.8300	39.1720
2	Catechol	3.32	1383.32	3676.20
3	Vanillic acid	6.11	9.01700	12.0820
4	Syringic acid	6.95	523.133	402.138
5	*p*- Coumaric acids	8.15	327.269	195.585
6	Ferulic acid	9.28	83.9982	256.731
7	*o*-Coumaric acid	9.49	50.9516	24.2010
8	Rosemarinic acid	13.1	138.940	476.256
9	Resveratrol	12.3	195.210	457.178
		**Flavonoids**		
1	Myricetin	12.84	267.18	257.347
2	Quercetin	14.73	13.066	112.354
3	Kaempferol	16.06	11.728	13.2910
4	Apigenin	16.19	9.7280	1.17700
		**Glycosides**		
1	Rutin	10.83	694.55	807.060
2	Mangiferin	11.96	3653.9	5955.35

Table S4

Food borne pathogen bacteria strains	Crystal violet (CV) stain O.D. at 570 nm		Inhibition %
	Control (Without MPEE)	Treated with 1000 µg/ml MPEE	
B. cereus ATCC 11778	0.80^{b}±0.30	0.01^{l}±0.02	98.75
S. aureus ATCC 5638	0.74^{c}±0.25	0.07^{j}±0.11	90.45
E. faecalis ATCC 7080	0.83^{a}±0.80	0.03^{k}±0.08	96.38
S. typhi DSM17058	0.71^{d}±0.49	0.10^{i}±0.08	85.92
E. coli ATCC 8739	0.54^{f}±0.62	0.20^{h}±0.12	62.96
S. sonnei DSM 5570	0.60^{e}±0.46	0.28^{g}±0.44	53.33

Values are means ± SD (*n* = 3). Data within all groups are analyzed using ANOVA by Duncan's test **(Duncan 1955)**.

Table S5

Compound	COX2	NFKB
Apigenin	-5.2	-6.3
Catechol	-3.5	-4.6
Coumarin	-4.2	-5.9
Ferulic	-3.9	-5.5
Gallic Acid	-3.7	-4.6
Kaempferol	-5.2	-6.3
Mangiferin	-5.0	-6.5
Myricetin	-5.0	-6.6
p_Coumaric	-3.9	-5.8
Quercetin	-5.0	-6.6
Resveratrol	-5.0	-6.7
Rosmarinic	-4.7	-7.1
Rutin	-5.5	-7.6
Syringic	-3.8	-4.5
Vanillic Acid	-3.7	-5.2

Table S6

Interaction	Amino acid	Type	Distance (A°)
**Conventional Hydrogen Bond**	ALA283:O	H-Bond	2.29406
**Pi-Pi Stacked**	A:TYR340	Hydrophobic	5.99677
**Amide-Pi Stacked**	(ALA287,ALA288)	Hydrophobic	4.53054
**Pi-Alkyl**	LEU284	Hydrophobic	5.08763
	ALA287	Hydrophobic	3.92755
	LEU253	Hydrophobic	5.22363
	LEU284	Hydrophobic	5.20631
	ALA287	Hydrophobic	4.06468
	ALA288	Hydrophobic	4.95132
	ALA283	Hydrophobic	5.45343

Table S7

Interaction	Amino acid	type	Distance
**Pi-Pi Stacked**	A:TRP65	Hydrophobic	4.00706
	A:TRP65	Hydrophobic	3.8472
	A:TRP65	Hydrophobic	4.89862
	A:TRP65	Hydrophobic	3.70811
**Pi-Alkyl**	A:PRO69	Hydrophobic	4.87344

Table S8

Interaction	Distance	Category	Type
**Interactions between Mangiferin with NFKB**			
:UNL1:H - A:TYR340:O	2.96784	Hydrogen Bond	Conventional Hydrogen Bond
:UNL1:H - A:GLY337:O	2.41143	Hydrogen Bond	Conventional Hydrogen Bond
A:TYR340:CB - :UNL1	3.60371	Hydrophobic	Pi-Sigma
A:TYR340 - :UNL1	5.39677	Hydrophobic	Pi-Pi T-shaped
A:TYR340 - :UNL1	4.78952	Hydrophobic	Pi-Pi T-shaped
A:TYR340 - :UNL1	5.32469	Hydrophobic	Pi-Pi T-shaped
:UNL1 - A:ALA283	4.98287	Hydrophobic	Pi-Alkyl
:UNL1 - A:ALA287	5.45628	Hydrophobic	Pi-Alkyl
:UNL1 - A:ALA283	5.0055	Hydrophobic	Pi-Alkyl
**Interactions between Mangiferin with COX2**			
A:TRP65 - :UNL1	3.82074	Hydrophobic	Pi-Pi Stacked
A:TRP65 - :UNL1	3.72038	Hydrophobic	Pi-Pi Stacked
A:TRP65 - :UNL1	4.9815	Hydrophobic	Pi-Pi Stacked
A:TRP65 - :UNL1	5.05174	Hydrophobic	Pi-Pi Stacked
A:TRP65 - :UNL1	3.8221	Hydrophobic	Pi-Pi Stacked
A:TRP65 - :UNL1	3.91535	Hydrophobic	Pi-Pi Stacked
A:TRP65 - :UNL1	3.82074	Hydrophobic	Pi-Pi Stacked

Table S9

Interaction	Distance	Type	Category
**Interactions between Bis(2-ethylhexyl) phthalate with NFKB**			
A:GLN215:HE21 - :UNL1:O	2.11718	Conventional Hydrogen Bond	Hydrogen Bond
A:GLN215:HE22 - :UNL1:O	2.51867	Conventional Hydrogen Bond	Hydrogen Bond
A:LEU253 - :UNL1	5.35288	Alkyl	Hydrophobic
A:LEU388 - :UNL1	5.33472	Alkyl	Hydrophobic
A:LEU391 - :UNL1	5.13178	Alkyl	Hydrophobic
:UNL1 - A:LEU211	5.36856	Pi-Alkyl	Hydrophobic
**Interactions between Bis(2-ethylhexyl) phthalate with COX2**			
UNL1:C - A:TRP65	3.62063	Pi-Sigma	Hydrophobic
A:LEU68 - :UNL1	4.76858	Alkyl	Hydrophobic
A:PRO69 - :UNL1	4.10029	Alkyl	Hydrophobic
A:TRP65 - :UNL1:C	4.47683	Pi-Alkyl	Hydrophobic

Supplementary Figure ligands

Fig. S1 Rutin **(A)**: 3D and **(B):** 2D poses interaction with NFKB.

Fig. S2 Rutin **(A):** 3D and **(B):** 2D poses interaction with COX2.

Fig. S3 Mangiferin 3D and 2D poses interaction with **(A)**: NFKB and **(B)** COX2.

Fig. S4 Bis(2-ethylhexyl) phthalate 3D and 2D poses interaction with **(A)**: NFKB and **(B)** COX2.

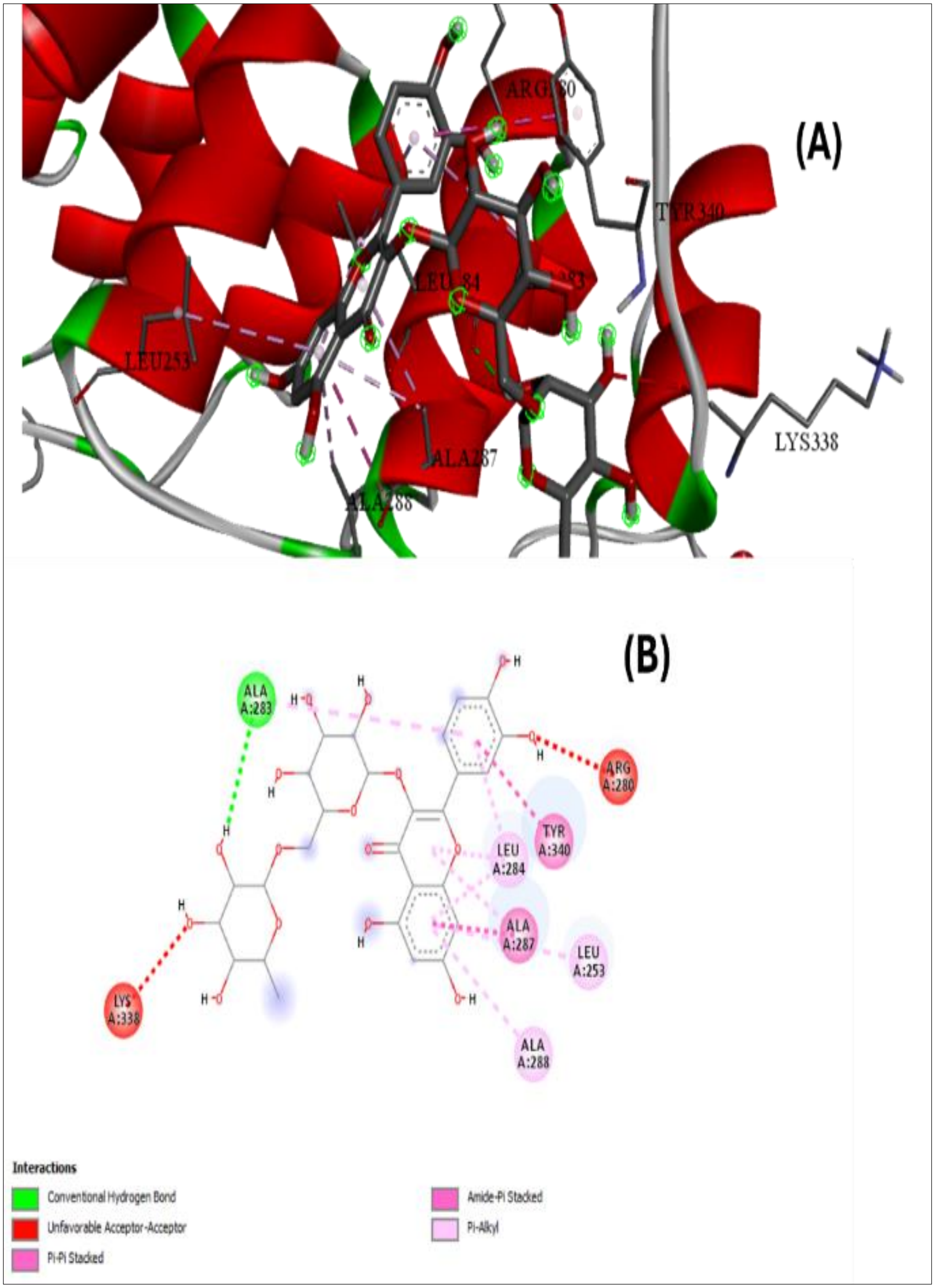
(A)
ARG280
TYR340
LEU284
LEU253
ALA287
ALA288
LYS338
(B)
ALA A:283
ARG A:280
TYR A:340
LEU A:284
ALA A:287
LEU A:253
LYS A:338
ALA A:288
Interactions
Conventional Hydrogen Bond
Unfavorable Acceptor-Acceptor
Pi-Pi Stacked
Amide-Pi Stacked
Pi-Alkyl

Fig. S1

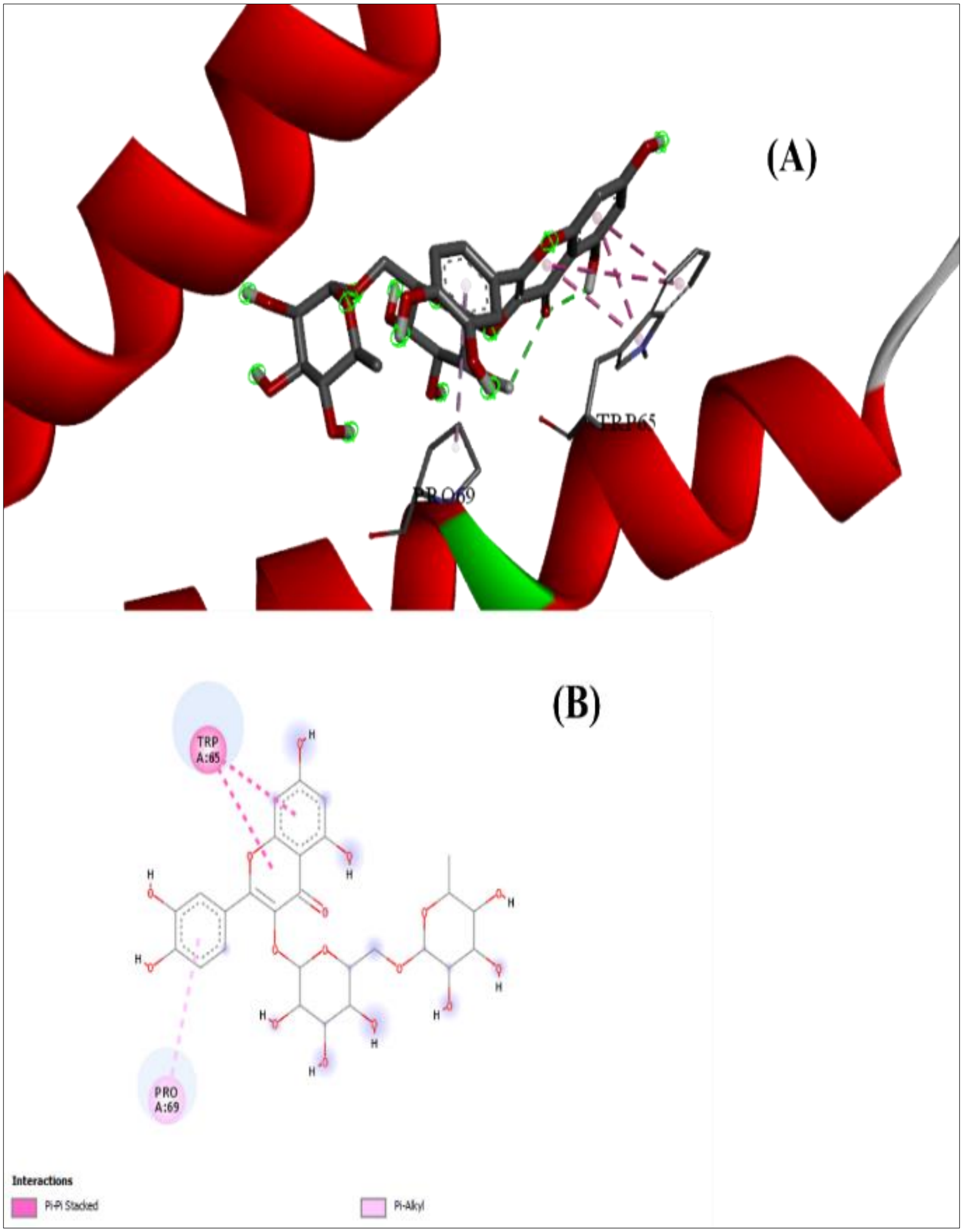
(A)
TRP65
PRO69
(B)
TRP A:65
PRO A:69
Interactions
Pi-Pi Stacked
Pi-Alkyl

Fig. S2

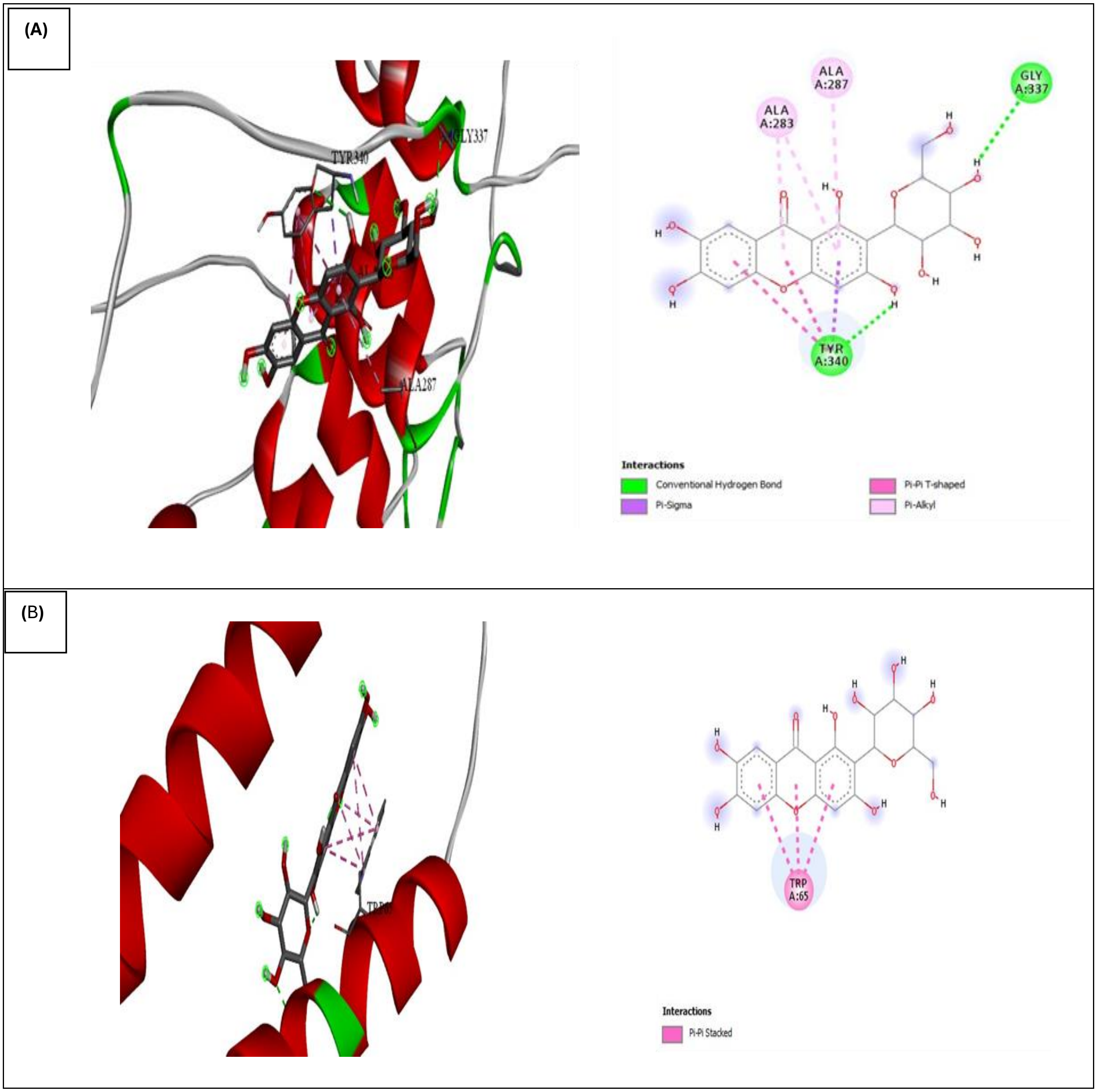

(A)
TYR340
GLY337
ALA287
ALA A:283
ALA A:287
GLY A:337
TYR A:340
Interactions
Conventional Hydrogen Bond
Pi-Sigma
Pi-Pi T-shaped
Pi-Alkyl
(B)
TRP A:65
Interactions
Pi-Pi Stacked

Fig. S3

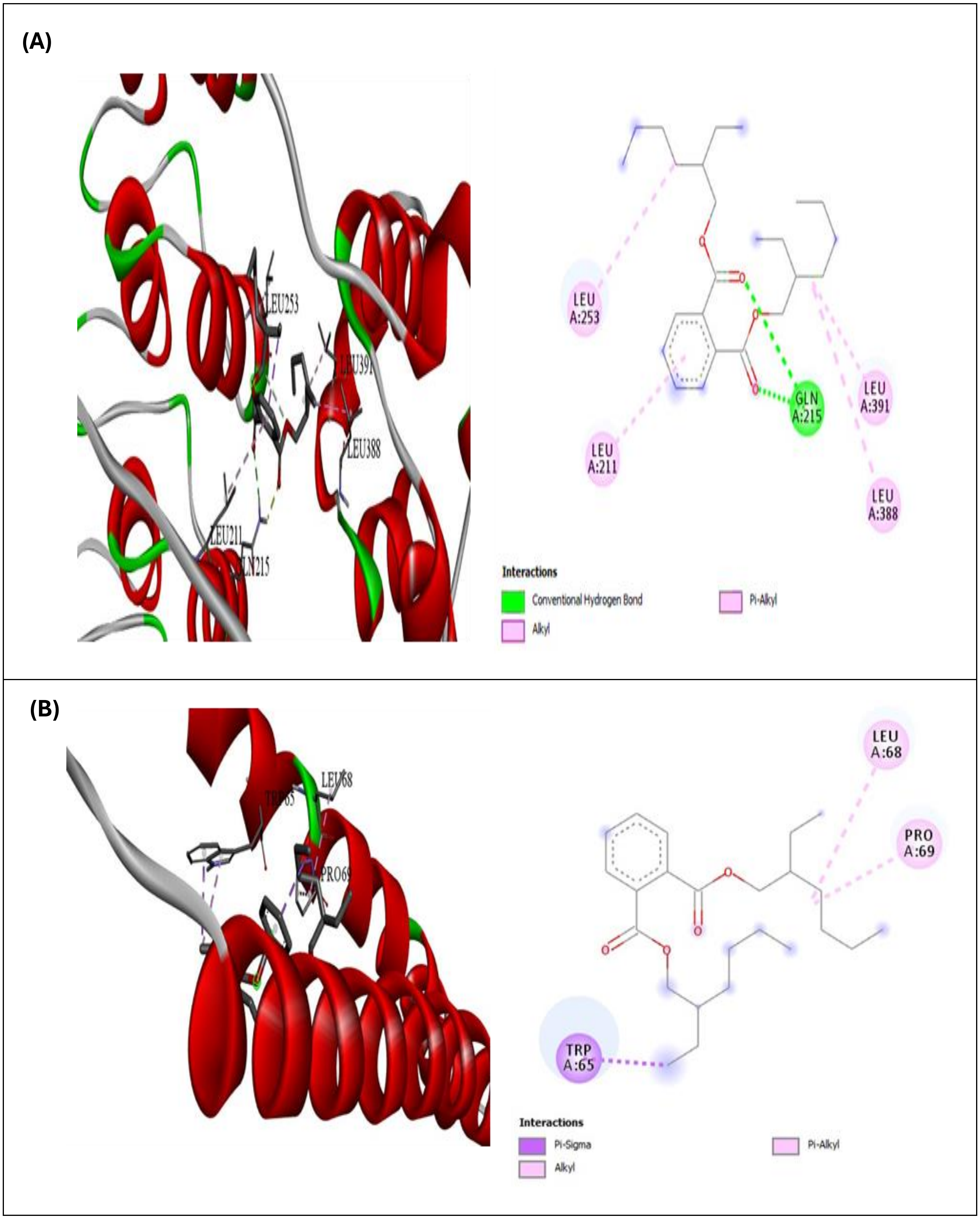
(A)
LEU253
LEU391
LEU388
LEU211
LEU
A:253
GLN
A:215
LEU
A:391
LEU
A:211
LEU
A:388
Interactions
Conventional Hydrogen Bond
Pi-Alkyl
Alkyl
(B)
PRO69
LEU
A:68
PRO
A:69
TRP
A:65
Interactions
Pi-Sigma
Pi-Alkyl
Alkyl

Fig. S4
